# 16. Attributable Mortality, Healthcare Costs and Out-of-Pocket Costs of *Clostridioides difficile* Infection in US Medicare Advantage Enrollees

**DOI:** 10.1093/ofid/ofab466.016

**Published:** 2021-12-04

**Authors:** Holly Yu, Jennifer L Nguyen, Tamuno Alfred, Jingying Zhou, Margaret A Olsen

**Affiliations:** 1 Heath Economics and Outcomes Research, Pfizer, Inc. Collegeville PA, Collegeville, PA; 2 Pfizer Inc., New York, New York; 3 Statistical Programming, Pfizer, Inc. New York NY, New York, NY; 4 Washington University in St. Louis, St. Louis, Missouri

## Abstract

**Background:**

US attributable CDI mortality and cost data are primarily from Medicare fee-for-service populations. Little is known about Medicare Advantage Enrollees (MAEs), who comprise about 39% of the Medicare population.

**Methods:**

Using 2017‒2019 Optum’s de-identified Clinformatics® Data Mart database, this retrospective cohort study identified first *C difficile* infection (CDI) episodes occurring in 2018 among eligible MAEs ≥66 y of age who were continuously enrolled for 12 mo before CDI diagnosis (baseline period). CDI was defined via ICD10 diagnosis codes or evidence of toxin testing with CDI antibiotic treatment. To assess all-cause mortality and CDI-associated healthcare and patient out-of-pocket (OOP) costs, CDI+ cases were matched 1:1 to CDI– controls using propensity scores (PS) and were followed through the earliest of death, disenrollment or end of the 12 mo followup. Additionally, outcome analyses were stratified by infection acquisition and hospitalization status.

**Results:**

Among 3,450,354 eligible MAEs, 15,195 (0.4%) had a CDI episode in 2018. Using PS generated from >60 variables collected in the baseline period, 14,928 CDI+ cases were matched to CDI– controls.

Over 12 mo of follow-up, the difference in 1-y attributable mortality was 7.9% in the CDI+ (26.3%) vs CDI– (18.4%) cohort (**Figure 1**). CDI-attributable mortality was higher among hospitalized CDI+ cases (18.4% for healthcare associated [HA]; 13.1% for community associated [CA]) vs nonhospitalized CDI+ cases (HA, 4.5%; CA, 1.0%).

Similarly, healthcare costs were higher for CDI+ vs CDI– patients, with excess mean total cost of &13,363 at the 2-mo follow-up (**Figure 2**). Total excess mean healthcare costs were greater among hospitalized CDI+ patients (HA, &28,139; CA, &28,136) than for nonhospitalized CDI+ patients (HA, &5741; CA, &2503). CDI-associated excess mean OOP cost was &409 for CDI+ cases at the 2 mo followup. Total excess mean OOP cost was highest in CA hospitalized CDI+ cases, followed by HA hospitalized CDI+ cases, HA nonhospitalized CDI+ cases and finally CA nonhospitalized CDI+ cases (&964, &574, &231 and &197, respectively).

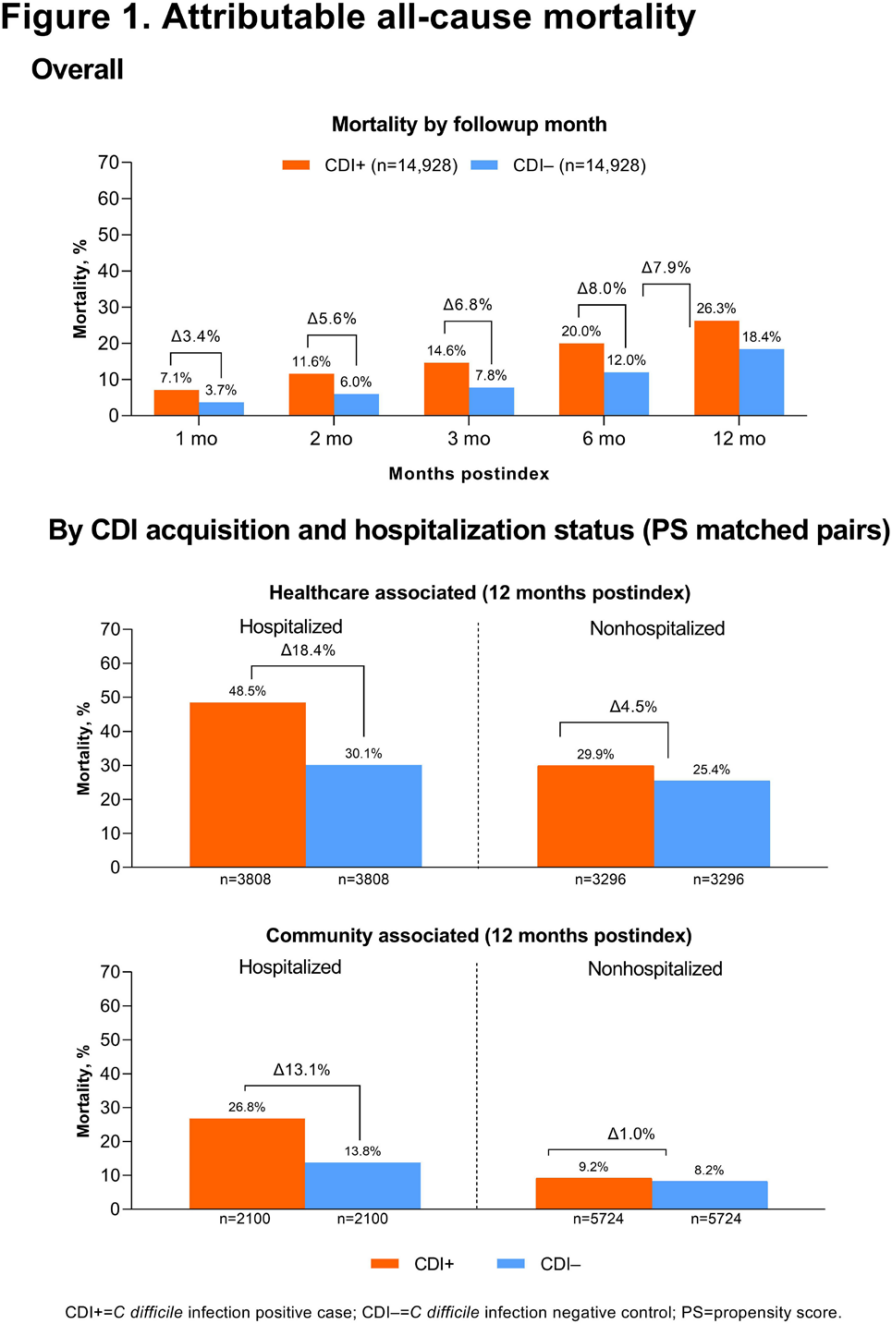

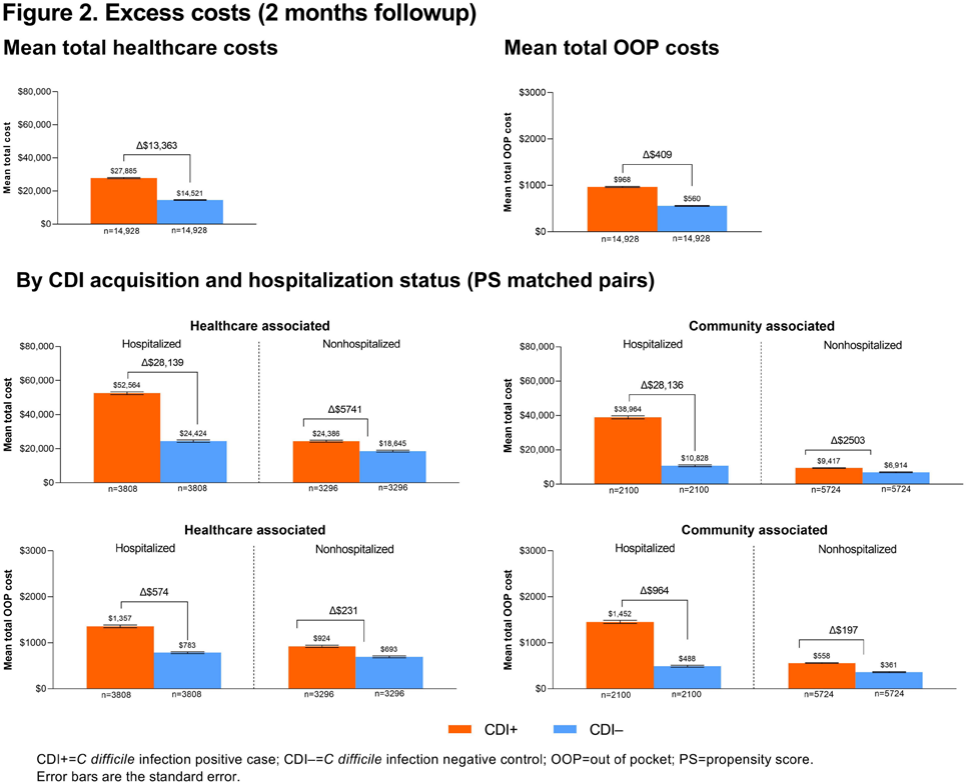

**Conclusion:**

CDI is associated with major mortality and total healthcare and OOP costs. Preventing CDI in the elderly may improve outcomes and reduce costs for healthcare systems and patients.

**Disclosures:**

**Holly Yu, MSPH**, **Pfizer Inc** (Employee, Shareholder) **Jennifer L Nguyen, ScD, MPH**, **Pfizer Inc.** (Employee) **Tamuno Alfred, PhD**, **Pfizer Inc.** (Employee) **Jingying Zhou, MA, MEd**, **Pfizer Inc** (Employee, Shareholder) **Margaret A. Olsen, PhD, MPH**, **Pfizer** (Consultant, Research Grant or Support)

